# Scalable Surveillance of E-Cigarette Products on Instagram and TikTok Using Computer Vision

**DOI:** 10.1093/ntr/ntad224

**Published:** 2023-11-08

**Authors:** Julia Vassey, Chris J Kennedy, Ho-Chun Herbert Chang, Ashley S Smith, Jennifer B Unger

**Affiliations:** Department of Population and Public Health Sciences, University of Southern California, Los Angeles, CA, USA; Center for Precision Psychiatry, Massachusetts General Hospital, Boston, MA, USA; Department of Psychiatry, Harvard Medical School, Boston, MA, USA; Department of Quantitative Social Science, Dartmouth College, Hanover, NH, USA; Information Sciences Institute, Viterbi School of Engineering, University of Southern California, Los Angeles, CA, USA; Department of Population and Public Health Sciences, University of Southern California, Los Angeles, CA, USA; Department of Population and Public Health Sciences, University of Southern California, Los Angeles, CA, USA

## Abstract

**Introduction:**

Instagram and TikTok, video-based social media platforms popular among adolescents, contain tobacco-related content despite the platforms’ policies prohibiting substance-related posts. Prior research identified themes in e-cigarette-related social media posts using qualitative or text-based machine learning methods. We developed an image-based computer vision model to identify e-cigarette products in social media images and videos.

**Aims and Methods:**

We created a data set of 6999 Instagram images labeled for 8 object classes: mod or pod devices, e-juice containers, packaging boxes, nicotine warning labels, e-juice flavors, e-cigarette brand names, and smoke clouds. We trained a DyHead object detection model using a Swin-Large backbone, evaluated the model’s performance on 20 Instagram and TikTok videos, and applied the model to 14 072 e-cigarette-related promotional TikTok videos (2019–2022; 10 276 485 frames).

**Results:**

The model achieved the following mean average precision scores on the image test set: e-juice container: 0.89; pod device: 0.67; mod device: 0.54; packaging box: 0.84; nicotine warning label: 0.86; e-cigarette brand name: 0.71; e-juice flavor name: 0.89; and smoke cloud: 0.46. The prevalence of pod devices in promotional TikTok videos increased by 15% from 2019 to 2022. The prevalence of e-juices increased by 33% from 2021 to 2022. The prevalence of e-juice flavor names and e-cigarette brand names increased by about 100% from 2019 to 2022.

**Conclusions:**

Deep learning-based object detection technology enables automated analysis of visual posts on social media. Our computer vision model can detect the presence of e-cigarettes products in images and videos, providing valuable surveillance data for tobacco regulatory science (TRS).

**Implications:**

Prior research identified themes in e-cigarette-related social media posts using qualitative or text-based machine learning methods. We developed an image-based computer vision model to identify e-cigarette products in social media images and videos. We trained a DyHead object detection model using a Swin-Large backbone, evaluated the model’s performance on 20 Instagram and TikTok videos featuring at least two e-cigarette objects, and applied the model to 14 072 e-cigarette-related promotional TikTok videos (2019–2022; 10 276 485 frames). The deep learning model can be used for automated, scalable surveillance of image- and video-based e-cigarette-related promotional content on social media, providing valuable data for TRS. Social media platforms could use computer vision to identify tobacco-related imagery and remove it promptly, which could reduce adolescents’ exposure to tobacco content online.

## Introduction

Exposure to tobacco-related content on social media is associated with adolescent e-cigarette use.^1–6^ Posts about e-cigarettes are the most prevalent type of tobacco-related posts on social media (eg, hashtag #vape on Instagram has over 30 million posts, which by far exceeds the number of posts related to cigars, cigarettes, or other tobacco products on this platform). Despite restrictions or bans on depicting and advertising tobacco-related content under community guidelines^7,8^^,^^9^on a variety of social media platforms, user-generated and promotional e-cigarette-related posts are still present on these platforms.^10,11^ Video-based platforms highly popular among youth (eg, TikTok) expose youth to tobacco content.^5,11–13^

The popularity of traditional text-based or multi-media platforms has been declining over the past decade (eg, 23% of adolescents use Twitter and 32% use Facebook), while popularity of video- and image-based platforms (Instagram and TikTok) has been increasing among adolescents (eg, 95% of them use YouTube, 67% use TikTok and 62% use Instagram).^14,15^ Short video clips on social media (eg, Instagram and TikTok) often have higher attentional engagement compared to still images^14^ and are also more popular among adolescents than longer videos, (eg, on YouTube).^16^ Research^12^ shows that adolescents had higher odds of e-cigarette lifetime and current (past 30-day) use and initiation if they frequently (several times a day) used TikTok compared to adolescents who used TikTok less frequently or not at all. Adolescents^12^ also had higher odds of e-cigarette lifetime use if they were frequently exposed (at least weekly) to tobacco-related content on TikTok compared to adolescents who were exposed to tobacco-related content on TikTok monthly or less frequently. E-cigarette use in TikTok and Instagram videos is often shown by general users or influencers in the context of a positive experience, humor, and jokes, and as a socially acceptable behavior.^11,17^ Based on the Prototype Willingness Model,^18,^19^^ people are more willing to engage in a behavior to the extent that they have a positive view of the prototypical person who performs that behavior.^5^ If adolescents observe friends, acquaintances, or influencers using e-cigarette products while appearing to be happy and popular on social media, they may see e-cigarette use as a behavior to emulate and perceive it as more normative and less risky.

Qualitative content analysis using human coders is common in identifying themes in tobacco-related images or videos on social media.^13,20,21^ One limitation of this method is the small sample size due to the time burden of review. Applying advanced computational methods is a valuable methodological contribution to tobacco control research, allowing for automated analysis of visual posts of big data-scale sample sizes. Advances in artificial intelligence methods, that is, machine learning (ML), can enhance current methods to analyze visual tobacco-related content. Only a handful of studies have applied machine learning to videos about tobacco products.^22^ For example, Kong et al.^23^ conducted deep learning—a subset of machine learning that uses artificial neural networks to mimic the learning process of the human brain—to examine how and which e-cigarette products are promoted to youth in YouTube videos. The study used video titles and descriptions as inputs to the bidirectional long short-term memory networks classification model. Vassey et al^24^ and Kennedy et al^25^ applied deep learning to classify e-cigarette products and nicotine warning labels in e-cigarette-related Instagram images. These studies^24,25^ used convolutional neural networks (CNN) which allow a model to gradually learn image features (eg, object shapes) in the process of image analysis and object detection. These neural networks (Inceptionv3, ResNet, and EfficientNet) were initially trained on 1000 classes of over 10 million images collected in a library ImageNet^26^ and were used as the basis for creating a customized image classifier capable of identifying e-cigarette products or nicotine warning labels in images. Although the original backbone model required training on such large data sets of images, the customized classifier—a fine-tuned version of the original model—was built on a smaller training sample size of images related to a specific research question (eg, recognition and classification of e-cigarette products). This downscaled training is based on the transfer learning method^27–29^ often used in deep learning models. Transfer learning adapts a model trained for one task to a new task by fine-tuning the original model. It allows the new model to maintain the knowledge obtained during its original training while learning new information in the process of retraining on a smaller data set, which does not require extensive training and computational power.^27^ The updated customized model is capable of highly accurate classifications. For example, the Inceptionv3 model used by Vassey et al.^24^ was retrained over 2–3 h on 1745 images featuring e-cigarette products and people’s faces. The model was trained to recognize six classes (ie, “man,” “woman,” “mod” [rebuildable and modifiable vaping devices], “pod” [refillable, rechargeable, or disposable vaping devices], “e-juice,” and “other”). The model achieved 0.90 validation accuracy, that is, was able to classify approximately 90% of the objects in the assigned classes correctly. In this study, we trained a DyHead (dynamic head framework)^30^ object detection model using a Swin–Large Transformer backbone. DyHead models are adaptable to different types of model architectures (eg, convolutional neural network or Transformer-based). Transformer-based architectures (or “transformers”) originated in modeling language understanding tasks (ie, natural language processing), but have been increasingly applied to object detection in images and videos.^31^ Unlike CNN that learn from repetition step by step, transformers look at all elements of a task at once and also pay the most attention to the important elements (eg, objects of interest in an image) and understand the relationships between different elements. This allows them to potentially be more accurate compared to CNN-based models.^31,32^

With the increasing popularity of video-based platforms like TikTok^52^, object detection in e-cigarette-related videos could be a valuable contribution to both computer vision and tobacco control research. Accurate computer vision models could be used by social media platforms to identify tobacco-related imagery and remove it promptly, which could reduce adolescents’ exposure to tobacco content online. Prior studies^33^ applied CNN-based deep learning models to analyze videos in different contexts (eg, detect aggressive driving). In this study, we conducted deep learning object detection (ie, identified multiple objects) in promotional e-cigarette-related videos posted by microinfluencers^34^ (brand ambassadors and content creators) on Instagram and TikTok. The objects included: mod and pod devices, e-juice containers, packaging boxes, smoke clouds, nicotine warning labels, e-cigarette brand names, and e-juice flavor names placed on e-cigarette products featured in videos. The main goals were (a) to evaluate the model’s performance on a validation set of social media videos, each 30 s long and featuring at least two e-cigarette-related objects in a single video; (b) to use the deep learning model to conduct e-cigarette object detection in e-cigarette-related promotional videos posted by microinfluencers on TikTok in 2019–2022. We assessed the prevalence of different e-cigarette objects and their temporal changes over the 4 years (2019–2022). We hypothesized that a pretrained object detection model fine-tuned on Instagram images featuring e-cigarette products would be able to detect e-cigarette-related objects in Instagram or TikTok videos.

## Method

### Data Collection

Using the social media data provider Meltwater,^35^ we accumulated prospectively and retrospectively 69 788 Instagram images and 38 376 Instagram and TikTok videos featuring e-cigarette products posted in 2019–2022 by over 200 United States based and international microinfluencers^34^ (eg, brand ambassadors and content creators) who promoted e-cigarettes on these platforms. The microinfluencers we tracked had over 1000 followers and a high user engagement rate (ratio of likes and comments to followers) of 1%–25% per post.^36^

### Supervised Deep Learning-related Procedures

**Figure 1.**
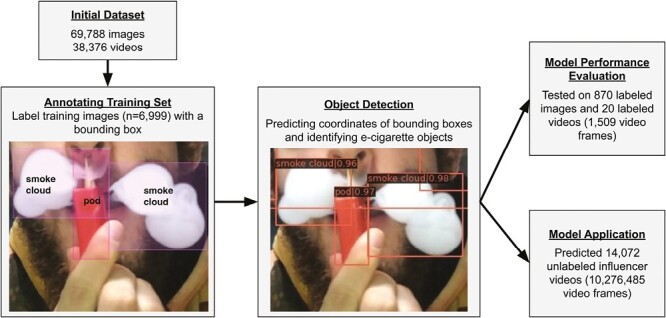
A flow chart of images and videos used for model training, evaluation and application. The figure includes an example of image annotation with bounding boxes (three e-cigarette-related objects were annotated: one pod device and two smoke clouds) and an example of image showing object detection with confidence scores (0.97 for a pod device and 0.96 and 0.98 for smoke clouds). Image source: the University of Southern California stock image repository.

The supervised deep learning-related procedures included:

(1) Image annotation with bounding boxes (Figure 1) using human coders;
(2) Training a deep learning model on the annotated images to:
  a) Predict the bounding box coordinates of e-cigarette objects (Figure 1);
  b) Predict an e-cigarette-related object inside each bounding box (ie, mod and pod devices, e-juice containers, packaging boxes, smoke clouds, as well as nicotine warning labels, e-cigarette brand names, and e-juice flavor names placed on e-cigarette products featured in images, Table 1 and Figure 1).
(3) Model performance evaluation on the annotated videos;
(4) E-cigarette object detection in new unlabeled videos (Figure 1).

**Table 1.** Objects to Identify in Images and Annotate with a Bounding Box

Object	Example	Description
1. Mod device	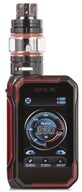	Big, bulky, often box-looking, rebuildable, modifiable e-cigarette (vape) devices for advanced vape users. Includes mod kits, boxes, atomizers, and accessories.
2. Pod device	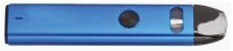	The newest generation of e-cigarette (vape) products, often for e-cigarette use (vaping) beginners. They are smaller and slicker than mods. Includes disposable, refillable, and rechargeable pod devices.
3. E-juice bottle	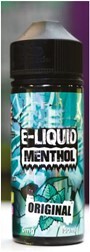	Bottles of nicotine or non-nicotine e-juices.
4. Box	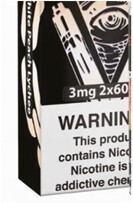	Packaging of e-juice, mod, or pod devices.
5. Smoke cloud	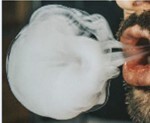	Exhaled smoke.
6. Nicotine warning label	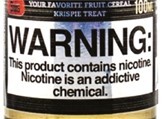	Warning label in an image/video or placed on an e-cigarette product featured in an image/video that contains the word “nicotine.”
7. E-juice flavor name	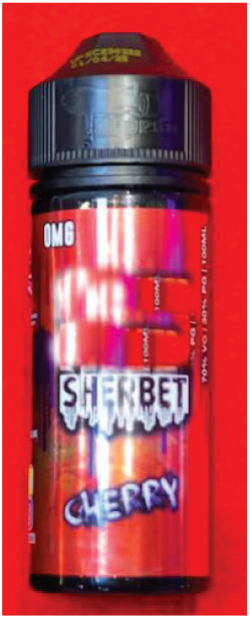	Presence of an e-juice flavor name (eg, sherbet cherry) on an e-cigarette product.
8. E-cigarette brand name	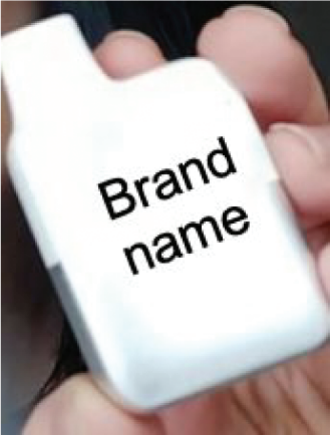	Presence of an e-cigarette brand name on an e-cigarette product.

Image source: the University of Southern California stock image repository.

Because the goal of this research was to identify all relevant objects in each video or image, we used an object detection model with a bounding box as opposed to an image classification model that only assigns a single label per image or video.^24^ A bounding box is a type of annotation used in computer vision that refers to a rectangle drawn around an object in an image or video. Bounding box deep learning models are typically composed of an object detector that identifies which pixels in an image or a video belong to an object and a regressor responsible for predicting the coordinates—a position—of the bounding box around that object (Figure 1). When multiple bounding boxes are present, multiple objects can be detected. Bounding box deep learning models require less training data, can be trained faster, and achieve better accuracy than traditional object detection models.^37^

### Image Annotation

A training sample of 6999 Instagram images featuring influencers promoting e-cigarette products was selected from the data set of images (*N* = 69 788) and annotated with a bounding box in the V7 Labs annotation application.^38^

To create an accurate training set, an annotator (trained by J.B.U., J.U., and C.J.K. who have tobacco control expertise) drew bounding boxes tightly around objects to ensure that the bounding box touched the edges of the object and that the object did not extend outside of the bounding box. (The detailed image annotation guideline is provided on GitHub, see Data Availability section). Two researchers (J.V. and C.J.K.) independently reviewed the labels to ensure consistency.

Because a computer vision model can analyze a video frame by frame as a sequence of images, a training sample based on images is appropriate to use for object detection in videos. Image labeling has several advantages compared to video labeling: (a) image labeling is less time-consuming; (b) using images makes it easier to create a relatively large training sample with a wide variety of e-cigarette objects.

We chose several e-cigarette product categories used in prior studies^24^ (ie, mod and pod devices and e-juices) and added new categories based on the observed e-cigarette-related attributes in images and videos in our data set (ie, smoke clouds, packaging boxes, warning labels, flavor, and brand names). We used V7 Labs software^38^ to label images, that is, draw bounding boxes over objects corresponding to the eight e-cigarette objects in Table 1. To decrease the rate of false positives, we included an additional sample of Instagram images with no e-cigarette products (*n* = 1175) to the training set: that is, images featuring a variety of objects unrelated to substance use (eg, makeup, food, and furniture). We did not annotate images in this additional training set with bounding boxes, but we tagged (labeled) them as “non-vaping.”

### Model Training

Model training was conducted in Python (version 3.8) using the MMDetection computer vision framework (version 2.28.2),^39^ an open-source object detection toolbox based on PyTorch (version 1.13).^40^ MMDetection has multiple well-known computer vision models built-in, which can readily be used for transfer learning as well as training custom models. MMDetection acts as a wrapper (assisting function) for training for a variety of object detection models, which reduces the person-time required to test alternative model architectures and conduct experiments.

We fine-tuned a DyHead^30^ object detector incorporating a Swin-Large pretrained backbone model (originally trained on over 10 million images of 1000 classes in the ImageNet library)^26^ on the 6999 e-cigarette-related images of eight classes (proportion of images per class: e-juice container: 19%; pod device: 11%; mod device: 7%; packaging box: 4%; nicotine warning label: 13%; e-cigarette brand name: 22%; e-juice flavor name: 16%; and smoke cloud: 8%) to customize the model to detect e-cigarette objects. Approximately 20% of the training set contained more than one type of object: all of them were annotated in an image. The DyHead and the Swin-Large model architecture were selected based on competitive performance on the COCO object detection leaderboard.^41^

The model was trained using 2 NVIDIA RTX A6000 GPUs for 12 epochs, with a batch size of two images, learning rate of 5e-5 with linear warm-up schedule (500 iterations), and step decay at epochs 8 and 11. The training was conducted with the AdamW optimizer with a weight decay of 0.05. Images were resized to a width of 2000 pixels and a height between 480 and 1200 pixels.

### Model Performance Evaluation

Model performance was evaluated on a 20% test set of the labeled images using the mean average precision (mAP) metric,^42^ which ranges from 0 (the lowest possible value) to 1 (the highest). The mAP is based on the following submetrics: recall (the proportion of true positives out of all positive predictions), precision (the proportion of true positives out of true positive and false negative predictions), confusion matrix (true/false positives and true/false negatives), and a confidence score,^42^ which mixes the object presence confidence and localization accuracy for a bounding box. We calculated mAP for each of the eight classes as the mean of average precisions across a set of 10 intersection-over-union (IoU) thresholds ranging from 0.5 to 0.95. A higher IoU threshold requires that the predicted bounding box coordinates are closer to the ground truth box coordinates (Supplementary Figure S1). mAP incorporates the trade-off between precision and recall, considering both false positives and false negatives. We also calculated class-specific positive predictive values (PPV) also known as precision for specific sensitivity (also known as recall) values on the image test (Table 2), using the bounding box overlap threshold (IoU) of 0.5.

**Table 2.** Class-specific Model Performance on the Image Test Set for Three E-cigarette Product Detection Scenarios

Object class	PPV at 80% Sensitivity	PPV at 50% Sensitivity	PPV at 20% Sensitivity
Mod device	49%	73%	81%
Pod device	72%	86%	89%
E-juice container	97%	97%	99%
Packaging box	94%	98%	100%
Nicotine warning label	97%	100%	100%
E-juice flavor	91%	97%	100%
E-cigarette brand name	85%	97%	98%
Smoke cloud	38%	82%	95%

PPV = positive predictive value (also known as precision). Sensitivity is also known as “recall.” Bounding box overlap threshold (IoU) = 50%.

We evaluated the model’s video performance on a validation set of 20 e-cigarette-related videos from Instagram and TikTok, featuring at least two e-cigarette-related objects and up to 30 s in duration. Videos were annotated with bounding boxes at five frames per second, which represents 1509 video frames.

The model was then applied to 14 072 unlabeled TikTok videos (posted in January 2019—August 2022) consisting of 10 276 485 total frames. The object detection confidence scores for each frame in the 14 072 videos were smoothed using a five-frame moving average (a series of averages of five-frame subsets of the full data set). Object classes with a maximum smoothed probability of 80% or greater were counted as present in a video. An earlier version of the customized deep learning model was used for the unlabeled data analysis. Additional data analysis and visualization was conducted in R version 4.2.2 (2022).

## Results

### Model Evaluation on Annotated Images and Videos

The model achieved an overall mean average precision (mAP) of 0.66 on the image test set. The class-specific mAP was: 0.89 for e-juice containers, 0.67 for pod devices, 0.54 for mod devices, 0.84 for packaging boxes, 0.86 for nicotine warning labels, 0.71 for e-cigarette brand names, 0.89 for flavor names, and 0.46 for smoke clouds. The class-specific PPV also known as precision for specific sensitivity (also known as recall) are shown in Table 2.

A total of 97% of the 1509 annotated video frames in the validation set contained e-cigarette-related objects. Of these, 100% were successfully detected by the model as containing e-cigarette-related objects. For the remaining frames, 100% were correctly identified by the model as having no relevant objects. The class-specific mean absolute errors (average absolute value of the difference between true counts and model predictions) for correct object counts were: 0.25 for mod devices, 0.32 for pod devices, 0.12 for e-juice bottles, 0.11 for boxes, 0.54 for smoke clouds, 0.18 for warning labels, 0.46 for e-cigarette brand names, and 0.29 for flavor names (Supplementary Video).

### Object Detection in Unlabeled Videos

**Figure 2.**
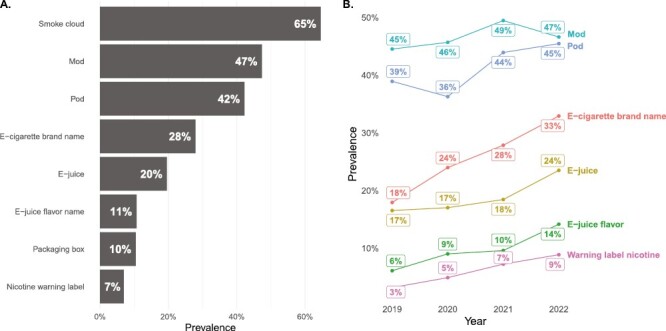
Prevalence and temporal trends of e-cigarette-related objects detected by the DyHead model in TikTok videos (*N* = 14 072) in 2019–2022.

The model performed object detection in 14 072 e-cigarette-related videos posted by 124 TikTok microinfluencers in 2019–2022. (There were 786 videos in 2019, 3423 in 2020, 5438 in 2021 and 4425 in 2022). Among the most prevalent e-cigarette-related objects were smoke clouds detected in 9091 (65%) videos, followed by mod devices detected in 6667 (47%) videos, and pod devices (including disposable devices) detected in 5949 (42%) videos. Warning labels were the least prevalent and were detected in 980 (7%) of the videos (Figure 2A).

From 2019 to 2022, the prevalence of mod devices in promotional TikTok videos remained relatively unchanged, while the prevalence of pod devices increased by 15%. The prevalence of e-juices was stable in 2019–2021, and it increased by 33% from 2021 to 2022. The prevalence of e-juice flavor names as well as e-cigarette brand names increased by about 100% from 2019 to 2022. Although the prevalence of nicotine warning labels increased by 200% from 2019 to 2022, its prevalence in TikTok videos in 2019 was low, only at 3%, and it remained low in 2022, at 9% (Figure 2B).

## Discussion

This study has important implications for tobacco regulatory science (TRS). We trained and validated a deep-learning model to conduct object detection (ie, identify one or more e-cigarette-related products) in promotional e-cigarette-related content posted by microinfluencers on TikTok and Instagram. Applying advanced computational methods such as deep learning is a valuable methodological contribution to tobacco control research allowing for automated analysis of visual posts of big data-scale sample sizes. We trained the customized deep learning model on Instagram images and evaluated its performance on a set of Instagram and TikTok videos. The evaluation results demonstrated the high accuracy of the model: detecting all e-cigarette-related objects in the annotated video frames, with the least mean absolute errors for e-juice bottles and packaging bottles and the highest mean absolute errors for smoke clouds.

Although collecting and labeling a training sample is time-consuming, running the trained and validated model on new videos or images (eg, a daily sample of 1000 social media posts) to detect e-cigarette products can be accomplished in an automated fashion, requiring no additional researcher time. To make image labeling more efficient, model-assisted annotation could be used,^43^ in which the current model is applied to unlabeled data, and those predicted bounding boxes are integrated into the annotation software. Human annotators would then only have to edit the preliminary bounding boxes generated by the models, rather than create all bounding boxes from scratch. As the model performance improves with larger training data, the accuracy of these “pre-annotations” would gradually increase, allowing human annotators to generate higher volumes of training labels in less time and with higher quality, further improving model performance.

We applied the model to unlabeled TikTok videos posted over 2019–2022 by 124 microinfluencers to assess the prevalence and changes in types of e-cigarette products promoted on TikTok over time. Almost half of the videos contained mod devices, followed by pod devices, whose presence in the videos increased in 2021–2022 from earlier years. Although the prevalence of the FDA (U.S. Food and Drug Administration)-mandated nicotine warning labels increased from 2019 to 2022, their presence was the least frequent compared with all the other e-cigarette-related objects detected by the deep learning model. This indicates that the influencer e-cigarette marketing remains largely noncompliant with the FDA marketing requirements.^44^ A large increase in the number of e-cigarette brand names and flavor names from 2019 to 2022 could indicate that e-cigarette promotional marketing has been growing on TikTok, which is consistent with a recent trend of a growing number of different brands (unrelated to e-cigarettes) actively promoting their content on TikTok.^34^

This study demonstrated that tobacco-related content remains poorly regulated on TikTok, one of the most-used social media among adolescents,^15^ despite the platform’s community guideline restricting or prohibiting displaying, promoting, or posting tobacco-related content.^45^ Especially concerning is that the number of pod devices, including disposables, popular among youth has been growing over the last several years. TikTok, or a third-party regulatory group, should more vigilantly monitor its substance-related content being posted in violation of the platform’s community guidelines.^45^ Such monitoring is essential for protecting youth from exposure to harmful posts. The removal of posts that violate the platform’s community guidelines could be accomplished through the flagging of suspected violations by content moderators.^11^ Studies^13^ show that while a small fraction of tobacco-related content gets removed from the platform, most of it remains available and accessible to youth, indicating that further improvement in the enforcement of community guidelines is necessary. In addition, federal authorities could regulate influencer e-cigarette-related marketing, because influencers are regarded as more trusted and authentic sources of content than traditional advertising^46^ and could diminish adolescents’ harmful perceptions of e-cigarettes promoted by influencers. Federal policymakers could penalize influencers for noncompliance with warning labels or sponsorship disclosure requirements or even require e-cigarette brands not to use influencers at all.^47^

Both short-form videos on entertainment-focused social media (ie, Instagram and TikTok) and longer-form videos on video-sharing platforms and streaming services (ie, YouTube, Twitch, and Netflix) have tobacco-related content.^10,11,21,36,48–50^ The deep learning model validated in this study on TikTok and Instagram videos could be used as a base model for e-cigarette or other tobacco product detection in other videos collected from a variety of visual online media. The model could be further adjusted to improve its prediction accuracy in different types of videos.^52^

This study is not only an important step in advancing computational methods and applying them to tobacco control research, but also an opportunity for disseminating information about these methods among TRS researchers. The model we trained is shared in an open repository for use by other researchers who are interested in applying similar classifications to different data sets. This will allow for quantifying tobacco content that TikTok and Instagram users (including youth) are being exposed to, which is a valuable addition to self-reported survey data often used to measure respondents’ exposure to tobacco content on social media.

### Limitations

Although the model we trained achieved high accuracy, it also misclassified some e-cigarette-related objects. Notably, the mean absolute error for smoke clouds was relatively high: 0.54. The amorphous shape of the smoke clouds, where the specific start and end region for a bounding box is ambiguous, makes it harder for a model to accurately predict the bounding box coordinates and detect the cloud inside the bounding box. Increasing the training sample size of images featuring a variety of smoke clouds as well as data augmentation (applying different transformations on the available data to synthesize new data and thus artificially increasing the training sample size) could contribute to higher model performance. The model sometimes assigned a false “mod” label to the so-called pod-mod systems, a fusion between box mod and pod devices, which should have been labeled as “pod” (the detailed image annotation guideline is available on GitHub, see Data Availability section). Although our training sample size was larger and more granular than in our previous study,^24^ an even larger and more varied training data set would further improve the accuracy of the model, particularly with regards to false positives on objects that resemble e-cigarette products. The model was not trained to identify more nuanced themes, for example, instructional versus promotional videos about e-cigarettes; such video classification-level object detection is being explored by the authors for future publications. The model was trained to detect and distinguish e-cigarette brand names from e-juice flavor names featured on e-cigarette products in videos, but it was not trained to perform scene text recognition (ie, to identify a specific e-cigarette brand name or e-juice flavor name, eg, banana ice). In addition, to improve performance and increase the speed of object detection, different model architectures could be considered. For example, YOLO^51^ architecture that enables end-to-end training and real-time speed or Contrastive Language-Image Pretraining models that allow for a smaller training sample and generalization on unseen objects without specifically training the model to detect these objects could be explored.

## Conclusion

The findings from this study demonstrated a successful application of a computer vision model for surveillance of e-cigarette-related objects in social media images and videos. Deep learning-based object detection provides automated analysis of visual posts in big data-scale sample sizes, offering efficiency compared to labor-intensive human coding (though still required to create the training data). The deep learning model assessed on the short Instagram and TikTok e-cigarette-related promotional content can be used as a base model to detect the presence of e-cigarettes and other tobacco products in long-form videos (eg, YouTube videos, Twitch streams, television shows, or films), providing valuable data for TRS. Social media platforms could use computer vision to identify tobacco-related imagery and remove it promptly, which could reduce adolescents’ exposure to tobacco content online.

Object detection, as well as audio detection, in videos in combination with automated analysis of text in captions or comments using various machine learning models, could complement each other in implementing a more nuanced automated classification and object detection tasks and could be directions for future research. Multimodal models (eg, applying deep learning to video, text, and sound components of visual social media posts) and scene text recognition (eg, identifying specific e-juice flavor names or e-cigarette brand names on e-juice containers or e-cigarette devices featured in images or videos) should also be explored as directions for future research. Last, knowledge about computational methods should be disseminated among tobacco control researchers by sharing deep learning detection and other machine learning models in open-access repositories.

## Supplementary Material

ntad224_suppl_Supplementary_Figures_S1

ntad224_suppl_Supplementary_Video

## Data Availability

The model, labeled training data, the annotation guideline, and the code underlying this article are shared on GitHub at https://github.com/e-cigarette-marketing-ml/e-cigarette-object-detection.
