# Supplementary material for: Scalable Surveillance of E-Cigarette Products on Instagram and TikTok Using Computer Vision
Source: Nicotine Tob Res. 2023 Nov 8;26(5):552–60. doi: 10.1093/ntr/ntad224 (PMC11033573; doi:10.1093/ntr/ntad224)

Ground truth

Prediction

Intersection over Union (IoU) = Overlap of Ground Truth & Prediction / Area of Union (Total Area)

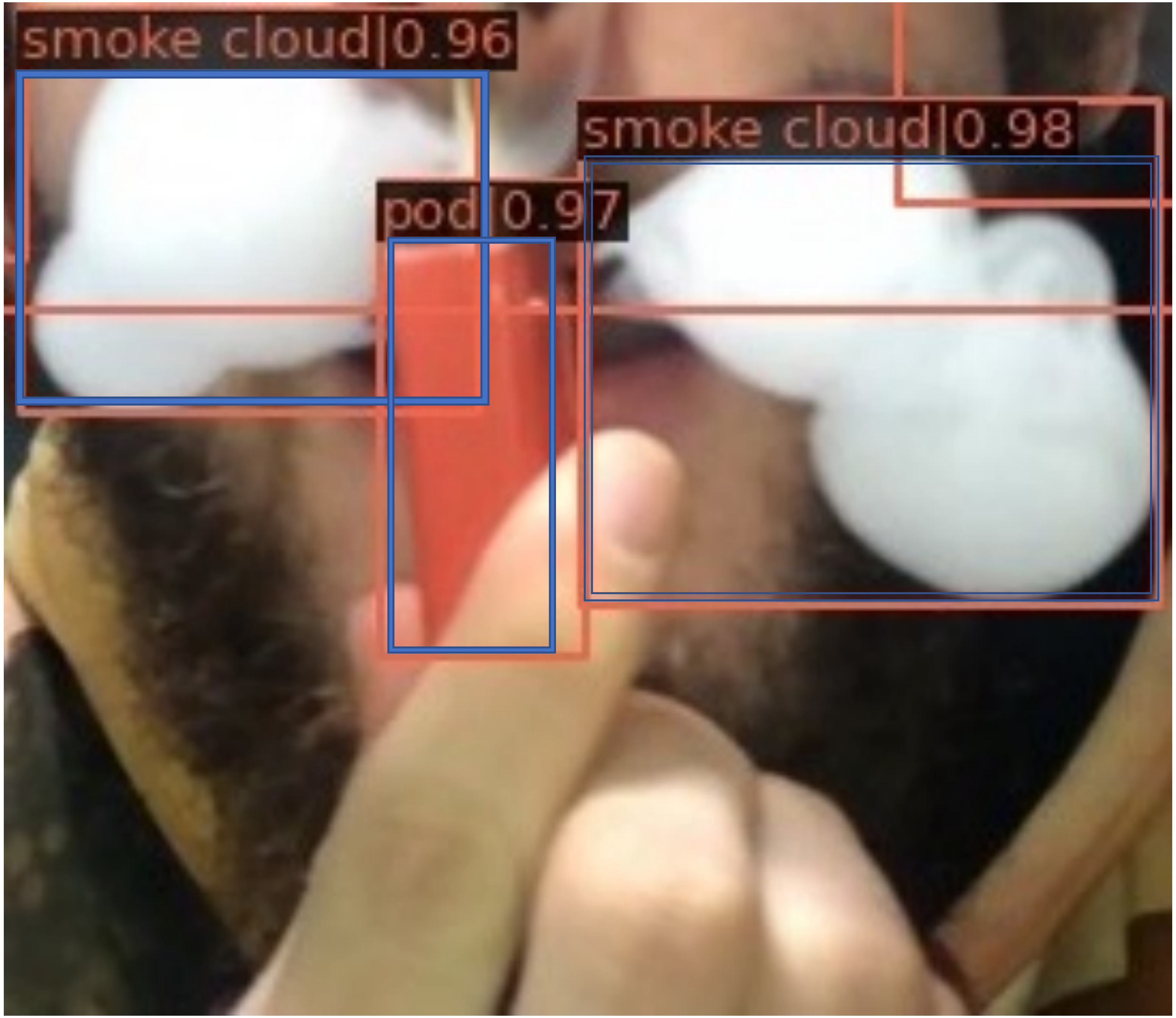

Supplement: ntad224_suppl_Supplementary_Figures_S1 [file ntad224_suppl_supplementary_figures_s1.pdf]
